# A Novel Phototransistor Device with Dual Active Layers Composited of CsPbBr_3_ and ZnO Quantum Dots

**DOI:** 10.3390/ma12081215

**Published:** 2019-04-13

**Authors:** Xu Zhang, Qing Li, Shikai Yan, Wei Lei, Jing Chen, Khan Qasim

**Affiliations:** 1 Joint International Research Laboratory of Information Display and Visualization, School of Electronic Science and Engineering, Southeast University, Nanjing 210096, China; 220161192@seu.edu.cn (X.Z.); 230179457@seu.edu.cn (S.Y.); lw@seu.edu.cn (W.L.); 101011529@seu.edu.cn (J.C.); 2 College of Electronic Science and Technology, Shenzhen University, Shenzhen 518061, China; qasimkhan@seu.edu.cn

**Keywords:** all inorganic perovskite quantum dots, ZnO QDs film, photodetector, transistor structure

## Abstract

Taking advantage of a large light absorption coefficient, long charge carrier diffusion length and low-cost solution processing, all-inorganic halides perovskite CsPbBr_3_ quantum dots (QDs) are combined with a ZnO QD film to construct a high-performance photodetector. In this work, a novel photodetector device based on transistor structure with dual active layers composed of CsPbBr_3_ and ZnO film is proposed. In this structure, CsPbBr_3_ film functions as the light-absorbing layer and ZnO film acts as the conducting layer. Owing to the high electron mobility and hole-blocking nature of the ZnO QDs film, the photo-induced electron-hole pairs can be separated efficiently. As a result, the device exhibits high performance with response of 43.5 A/W, high detection up to 5.02 × 10^11^ Jones and on/off ratio of 5.6 × 10^4^ under 365 nm light illumination. Compared with the ZnO-only phototransistor (the photodetector with the structure of transistor) the performance of the CsPbBr_3_ phototransistor showed significant improvement, which is superior to the majority of photodetectors prepared by perovskite. This work demonstrates that the ZnO QDs film can be applied in the photodetector device as a functional conducting layer, and we believe that the hybrid CsPbBr_3_/ZnO phototransistor would promote the development of low-cost and high-performance photodetectors.

## 1. Introduction

Lead halide perovskites have attracted extensive attention in photoelectronic applications due to the large absorption coefficient [1], high charge carrier mobility [2,3], and large diffusion length [4,5,6]. The power conversion efficiency of the solar cells consisting of organic–inorganic hybrid perovskite has already recently achieved 23.7% [7]. The external quantum efficiency (EQE) of the light emitting diodes (LEDs) based on lead halide perovskites has been improved from 0.76% to 20.3% in a short period [8,9,10,11,12]. There are also many researches on the photodetectors based on halide perovskites. Dou et al. demonstrated a solution-processed hybrid perovskite photodetector with a large detection approaching 10^14^ Jones [13]. Deng et al. reported flexible and semitransparent CH_3_NH_3_PbI_3_ network photodetector. The device shows a response of 0.1 A/W and high detection up to 1.02 × 10^12^ Jones [14]. Yu et al. reported a self-powered photodetector based on a ZnO/CH_3_NH_3_PbI_3_ heterojunction. The device exhibits high performance with response of 24.3 A/W, high detection up to 3.56 × 10^14^ Jones [15]. However, these reports focus less on the all-inorganic perovskites. Different from the organic–inorganic hybrid perovskites, all-inorganic perovskites CsPbX_3_ (X = Cl, Br, I) own higher stability in moist environments [16,17]. In addition, the all-inorganic perovskites QDs have high PL quantum yield (more than 90% QY for green PL from CsPbBr_3_ QDs), large absorption coefficient and narrow FWHM [18,19]. Therefore, the all-inorganic perovskites QD is a promising candidate for the construction of high-performance and high stable photodetector. However, the carriers transport properties of the all-inorganic perovskite photodetector are inferior compared to the photodetector based on organic–inorganic hybrid perovskites [20,21]. In order to accelerate the separation and transport of photo-induced electron-hole pairs, several two-dimensional materials have been integrated with all-inorganic perovskites, such as graphene, WS_2_, and MoS_2_ [22,23,24]. However, these two-dimensional materials increase the dark state current of the photodetector, which is an obstacle for improving the on/off ratio of the photodetector [25]. Therefore, it is imperative to develop suitable functional materials to combine with all-inorganic perovskite absorption layer.

The nontoxic solution-processed ZnO QDs with a direct wide bandgap of 3.37 eV and large exciton binding energy of 60 meV have been studied by many researchers [26,27]. They have been used as the emitting layer in ultraviolet QLEDs with the near-band-edge (NBE) emission [28,29,30,31,32]. Furthermore, the ZnO QDs have been widely used as the electron transport layer in QLEDs and solar cells due to the high electron mobility (2 × 10^−3^ cm^2^/V·s) [33,34,35]. Besides, ZnO QDs have higher thermal stability and reduced sensitivity to oxygen and moisture compared with the organic materials [36]. Pan et al. demonstrated the size tunability of sol-gel synthesized ZnO QDs in the electron transport layer in QLEDs. With the 2.9 nm sized ZnO, the best performance of the device is achieved with a maximum current efficiency of 12.5 cd/A and EQE of 4.2% [35]. Tavakoli et al. reported that a quasi-core/shell structure of ZnO/reduced graphene oxide (rGO) QDs was synthesized and employed as an electron transport layer in the fabrication of cell solar. The perovskite solar cell exhibited power conversion efficiency as high as 15.2% [37]. Due to the high electron mobility and appropriate energy band alignment, ZnO QDs is suitable functional material for the fabrication of photodetector based on all-inorganic perovskite QDs. 

In this paper, we have fabricated a photodetector device based on CsPbBr_3_ and ZnO QDs film as dual active layers of phototransistor. Because of the large absorption coefficient and long diffusion lengths of charge carriers in perovskites, as well as the high electron mobility of ZnO, the photo-induced carriers can be separated efficiently, employing ZnO film as a transport layer. As a consequence, this device has exhibited a high response of 43.5 A/W, a high detection up to 5.02 × 10^11^ Jones and an on/off ratio of 5.6 × 10^4^ under 365 nm light illumination with the intensity of 3 mW/cm^2^. Compared with the phototransistor without ZnO quantum dot films and the phototransistor without CsPbBr_3_ QDs film, the performance of the device is significantly improved, which is superior to most photodetectors prepared from perovskite materials.

## 2. Materials and Methods 

### 2.1. Preparation of CsPbBr_3_ QDs

**Figure 1:**
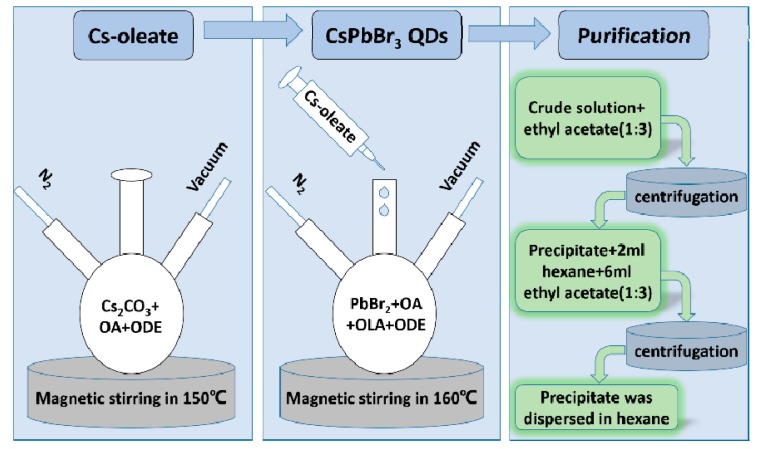
Preparation steps of CsPbBr_3_ QDs.

The CsPbBr_3_ QDs were synthetized as shown in Figure 1 using the hot-injection method reported by Kovalenko and co-workers with some modification [18]. Firstly, it is the preparation of Cs-oleate. Cs_2_CO_3_ (3.256 g, Aladdin, 99.9%, Shanghai, China) was loaded into 500 mL 3-neck flask along with octadecene (ODE, 160 mL, Aladdin, 90%) and oleic acid (OA, 10 mL, Aladdin, 90%), dried under vacuum for 1 h at 120 °C, and then heated under nitrogen atmosphere to 150 °C until all Cs_2_CO_3_ reacted with OA. Secondly, it is the synthesis of CsPbBr_3_ QDs. ODE (100 mL) and PbBr_2_ (1.38 g, Aladdin, 99.0%) were loaded into 500 mL three-neck flask and dried under vacuum for 1 h at 120 °C. Dried oleylamine (OLA, 10 mL, Aladdin, 90%) and dried OA (10 mL) were injected at 120 °C under nitrogen atmosphere. When PbBr_2_ salt was complete dissolved, the temperature was raised to 160 °C and Cs-oleate solution (more than 8 mL, ensure the PbBr_2_ salt to react completely) was quickly injected and, 5 s later, the mixture was bathed in ice water. Finally, the CsPbBr_3_ QDs were purified using the method reported by Zeng and co-workers [11]. First, ethyl acetate was mixed with the crude solution with a volume ratio of 3:1, the precipitate was dispersed in 2 mL of 1-octane or hexane after centrifugation. Second, the solution was centrifuged after 6 mL ethyl acetate added into the 2-mL hexane dispersion. Finally, the precipitate was dispersed in 1-octane or hexane with a certain concentration.

### 2.2. Preparation of ZnO QDs

**Figure 2:**
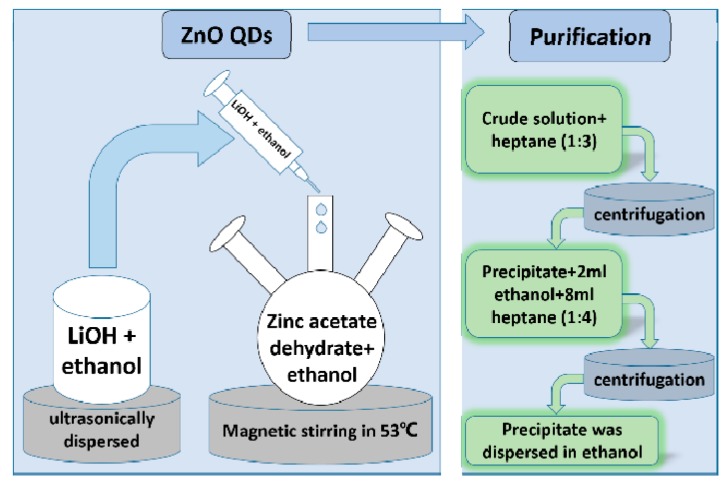
Preparation steps of ZnO QDs.

The ZnO QDs were synthesized as shown in Figure 2 using the same method as our previous reports [28,29,30,31]. Firstly, zinc acetate dihydrate (2.195 g) was added into 500 mL three-neck flask containing 100 mL ethanol at 70 °C, LiOH (13.8 mmol, 0.580 g) was dispersed in 100 mL ethanol at 50 °C. A reaction between these two solutions was initiated by mixing the two solutions under vigorous stirring, and keeping it at 53 °C for variable time to grow ZnO QDs with different sizes. Secondly, heptane was added into the synthesized solution with a volume ratio of 3:1, and the precipitate was dispersed in 2 mL ethanol after centrifugation at 10000 rpm. Then the mixed solution was centrifuged after adding 8 mL heptane. Finally, the precipitate was dispersed in ethanol in the desired concentration.

### 2.3. Fabrication of the Phototransistor

**Figure 3:**
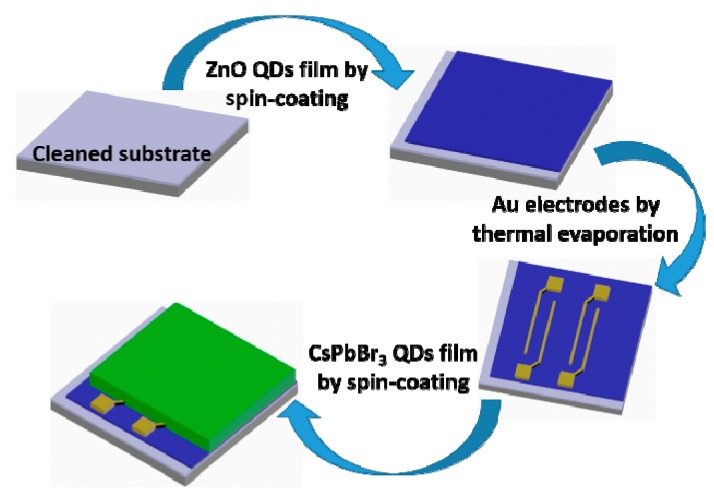
Fabrication steps of the phototransistor.

The fabrication steps of the phototransistor with the dual active layer is shown as Figure 3. The substrate is heavily p-doped Si with 300 nm SiO_2_. The substrate was cleaned with water, acetone, isopropanol with successive ultrasonic treatment, and followed by the UV–ozone treatment for 30 min. Firstly, the ZnO QDs solution (25 mg/mL) was spin-coated at 2000 rpm for 30 s, and then annealed at 140 °C for 20 min. Au source and drain electrodes were deposited on ZnO QDs layer by thermal evaporation with a shadow mask. The channel length and width were 20 μm and 100 μm. Then, the CsPBr_3_ QDs (20 mg/mL) were spin-coated on the top of ZnO/Au layer at 1400 rpm for 30 s, and annealed at 100 °C for 20 min. The devices with the single layer (ZnO QDs or CsPbBr_3_ QDs) as the reference devices were fabricated with the same method and condition. 

## 3. Results and Discussion

**Figure 4:**
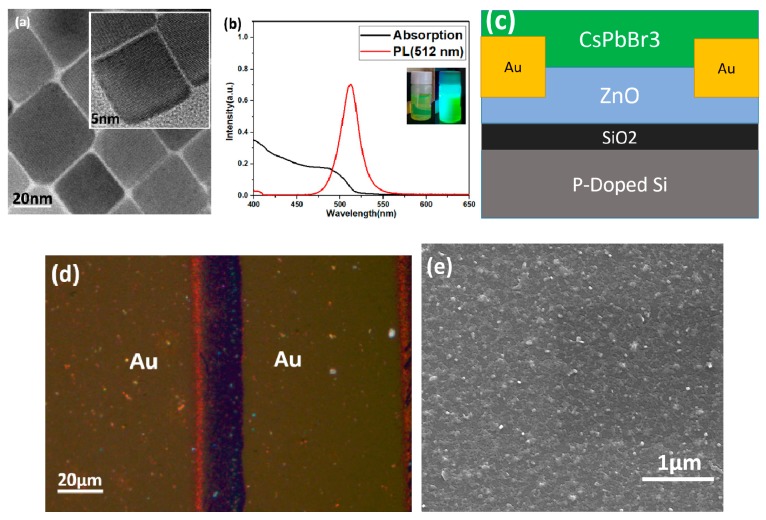
(**a**) Low-resolution TEM image of the CsPbBr_3_ QDs. [Inset: the corresponding high-resolution image]. (**b**) The absorption and PL spectra of the CsPbBr_3_ QDs. [Insert: the photographs of CsPbBr_3_ QD without (left) and with (right) 365 nm light excitation]. (**c**) Schematic illustration of the phototransistor. (**d**) Optical image of the phototransistor. (e) SEM image of CsPbBr_3_ QDs film on ZnO QDs film.

Figure 4a shows the TEM image for the as-prepared CsPbBr_3_ QDs. The image reveals pure cubic crystalline form of the QDs which is in good agreement with the previous literature [6,18]. The corresponding high-resolution images are shown in the insert of Figure 4a. Figure 4b shows the absorption and PL spectra of CsPbBr_3_ QDs. The optical band gap of CsPbBr_3_ QDs was estimated to be 2.38 eV, and the corresponding FWHM is 24.5 nm. The insert shows the photographs of CsPbBr_3_ QDs without (left) and with (right) 365 nm light excitation, which demonstrates the QDs have high PL intensity. The schematic illustration of the phototransistor is shown in Figure 4c, the ZnO QDs film and CsPbBr_3_ QDs film were fabricated by spin-coating and anneal method. Figure 4d shows the corresponding optical image of the phototransistor, the length and width of the channel between two gold electrodes were 20 μm and 100 μm, respectively. Figure 4e is the SEM image of CsPbBr_3_ QDs film on ZnO QDs film, which indicates that CsPbBr_3_ QDs have formed a dense layer of film. 

**Figure 5:**
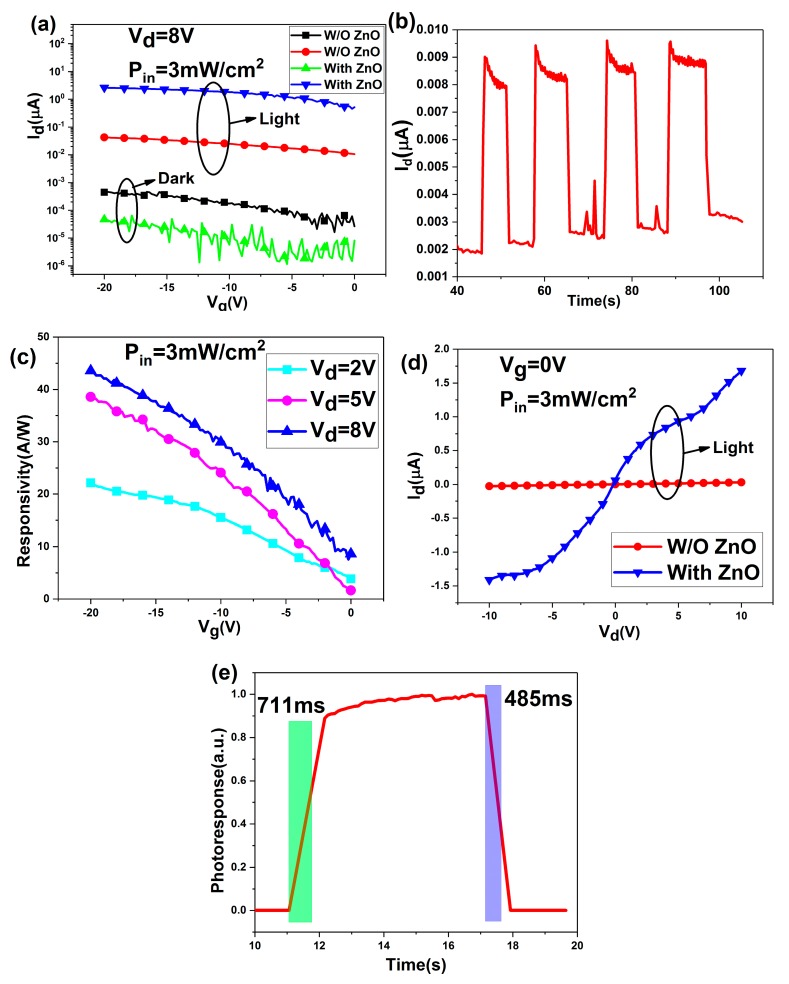
(**a**) Transfer characteristic curves of with and without ZnO QDs film phototransistors with and without 365 nm LED illumination (3 mW/cm^2^). Vd = 8 V. (**b**) The I-T curve of the device with single ZnO QDs film with 365 nm LED illumination (3 mW/cm^2^), V_g_ =-20 V, V_d_ = 8 V. (**c**) The response of the With ZnO device as a function of V_g_ and V_d_. (**d**) Output characteristic curves of with and without ZnO QDs film phototransistors with 365 nm LED illumination (3 mW/cm^2^), V_g_ = 0V. (**e**) Photoresponse curve for rise and decay time.

Figure 5a shows the transfer characteristic of the phototransistors. Both the devices of with ZnO QDs film (With ZnO) and without ZnO QDs film (W/O ZnO) exhibit obvious p-type semiconducting properties. The drain current (I_d_) can be modulated by gate voltage (V_g_). With the introduction of ZnO QDs film, the photocurrent greatly increased from 0.042 μA to 2.61 μA (V_g_ = −20 V, drain voltage (V_d_) = 8 V) under the illumination of 365 nm LED with the intensity of 3 mW/cm^2^. The result can be explained that in the W/O ZnO device, the photo-induced electron-hole pairs generated in CsPbBr_3_ QDs film are unable to separate efficiently the photo-excited electrons due to the large barrier of 1.7 eV between CsPbBr_3_ QDs and Au electrode. However, the With-ZnO device benefited from the small barrier of 1 eV between CsPbBr_3_ QDs and ZnO QDs, the photo-induced electrons are transferred to ZnO QDs film readily under the built-in field, and the high electron mobility of ZnO accelerates the transfer of the electrons to electrode. In order to verify, the device with single ZnO QDs film was fabricated. Figure 5b shows the I-T curve of the device (V_g_ = −20 V, V_d_ = 8 V) under the illumination of 365 nm source with the intensity of 3 mW/cm^2^, the device has a low on/off ratio of 4, and the photocurrent is also very small. The result can help to exclude the possibility that the electron–hole pairs are generated in ZnO QDs film. Therefore, increasing of photocurrent from 0.042 μA to 2.61 μA should attribute to the introduction of ZnO, which accelerates the separation and transfer of the photo-induced electron-hole pairs generated in CsPbBr_3_ QDs film. The dark-current of the With ZnO phototransistor is 0.047 nA, which is smaller than that of the W/O ZnO phototransistor, which attribute to that the introduction of ZnO QDs film increases the resistivity of the device. Figure 5c shows the response of the With ZnO device as a function of V_g_ and V_d_. The With-ZnO phototransistor exhibited a high response (R) of 43.5 A/W and an on/off ratio of 5.6 × 10^4^ under 365 nm light illumination, which are enhanced for 62-fold and 560-fold compared with the W/O ZnO phototransistor. Besides, we evaluated the specific detection (D*), the value can be estimated to be 5.02 × 10^11^ Jones (V_g_ = −20 V, V_d_ =8 V). The response and the detection of the photedetector were calculated according to the following relations [21,38]:

(1) $$R=\frac{\left|I_{d,light}|-|I_{d,dark}\right|}{P_{in}\times L\times W}$$

(2) $$D^{*}={\frac{R\times (L\times W)}{{\left(2e\times I_{d,dark}\right)}^{\frac{1}{2}}}}^{\frac{1}{2}}$$

where *L* and *W* are the channel length and width of the phototransistor respectively, *P_in_* = 3 mW/cm^2^, *e* is the electron charge, and the performance is better than most prepared perovskite photodetectors, Table 1 shows the performance comparisons of the prepared perovskite photodetectors.

Figure 5d shows the output characteristics of the phototransistors with the light illumination. The result demonstrates that the introduction of ZnO QDs film can accelerate the separation and transfer of the photo-induced electron-hole pairs as described above, and the output characteristics show linear dependence on the drain voltage with 365 nm light illumination, V_g_ = 0 V. This indicates an ohmic contact between the Au electrodes and the films [39]. 

Figure 5e shows the typical photoresponse curve for rise and decay time of the With ZnO phototransistor. The on/off switching behavior was repeated over multiple cycles, indicating the robustness and reproducibility of the phototransistors. The rise time and decay time were defined as the time rise to (1-1/e) of the maximum photocurrent from the dark current and recovery to 1/e of the maximum photocurrent, respectively [20]. The rise and decay time of the With ZnO phototransistor were determined to be 711 ms and 485 ms, respectively. 

**Figure 6:**
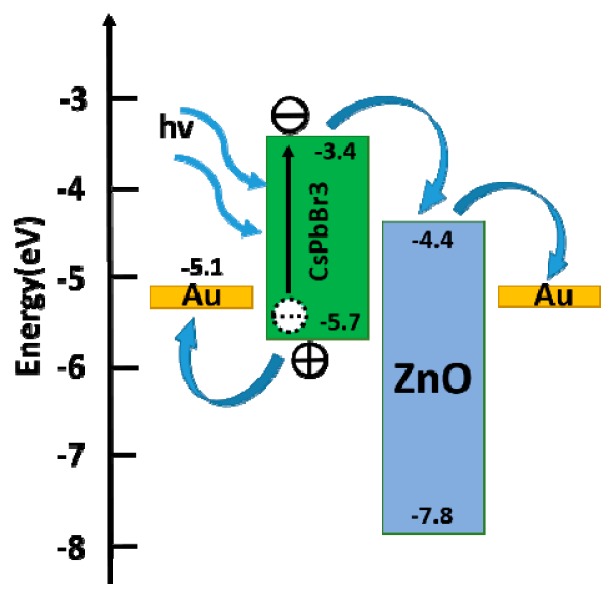
Schematic illustration of the energy level of the complete phototransistor.

Figure 6 shows the Schematic illustration of the energy level of With ZnO phototransistor. Electron-hole pairs are generated in the CsPbBr_3_ QDs film under 365 nm light irradiation. Benefited from the small barrier of 1eV between CsPbBr_3_ QDs and ZnO QDs, the electrons are transferred to ZnO QDs film readily under the built-in field. The high electrons mobility of ZnO QDs film accelerates the electrons transferring to electrode, while the holes were left in the valence band of CsPbBr_3_ QDs film [20,21,39]. Therefore the introduction of ZnO QDs film results in the high photoresponse of the detector.

## 4. Conclusions

The novel phototransistor based on CsPbBr_3_ QDs and ZnO QDs has been fabricated using a low-cost solution processing method. CsPbBr_3_ QDs film is used as the light absorption layer with large absorption coefficient, and ZnO QDs film is used as the carrier transport channel due to the high electron mobility. The introduction of ZnO QDs film decreases the barrier for transferring of electrons and accelerates the generated electrons transferring to electrode. The phototransistor based on CsPbBr_3_ QDs and ZnO QDs exhibited a high response of 43.5 A/W, high detection up to 5.02 × 10^11^ Jones and on/off ratio of 5.6 × 10^4^ under 365 nm light illumination with the intensity of 3 mW/cm^2^. The response and the on/off ratio are enhanced for 62-fold and 560-fold compared with the phototransistor without ZnO QDs film, respectively. This work demonstrate that the ZnO QDs film is a promising carrier transport material, and the phototransistor based on CsPbBr_3_ QDs and ZnO QDs fabricated using a low-cost solution processed method can be a promising candidate for high-performance photodetectors.

## Figures and Tables

**Table 1:** Performance comparisons of the prepared perovskite photodetectors.

Materials	Response (A/W)	On/off Ratio	Ref.
CsPbBr_3_ QDs	0.005	10^5^	[16]
CsPbBr_3_ Micro-particles	0.18	10^3^	[40]
MAPb(Br/I)_3_ thin films	0.055	10^2^	[41]
CsPbBr_3_-ZnO films	4.25	10^4^	[21]
MAPbI_3_-ZnO Nanorod	24.3	/	[15]
CsPbBr_3_-ZnO QDs	43.5	10^4^	This work
